# Bench to bedside review: therapeutic modulation of nitric oxide in sepsis—an update

**DOI:** 10.1186/s40635-019-0274-x

**Published:** 2019-12-02

**Authors:** Simon Lambden

**Affiliations:** Department of Medicine, Addenbrooke’s Hospital, Cambridge University, 5th Floor, Cambridge, CB20QQ UK

**Keywords:** Nitric oxide, Sepsis, Septic Shock, Arginine, Asymmetric dimethylarginine, Citrulline, Tetrahydrobiopterin

## Abstract

Nitric oxide is a signalling molecule with an extensive range of functions in both health and disease. Discovered in the 1980s through work that earned the Nobel prize, nitric oxide is an essential factor in regulating cardiovascular, immune, neurological and haematological function in normal homeostasis and in response to infection. Early work implicated exaggerated nitric oxide synthesis as a potentially important driver of septic shock; however, attempts to modulate production through global inhibition of nitric oxide synthase were associated with increased mortality. Subsequent work has shown that regulation of nitric oxide production is determined by numerous factors including substrate and co-factor availability and expression of endogenous regulators. In sepsis, nitric oxide synthesis is dysregulated with exaggerated production leading to cardiovascular dysfunction, bioenergetic failure and cellular toxicity whilst at the same time impaired microvascular function may be driven in part by reduced nitric oxide synthesis by the endothelium. This bench to bedside review summarises our current understanding of the ways in which nitric oxide production is regulated on a tissue and cellular level before discussing progress in translating these observations into novel therapeutic strategies for patients with sepsis.

## Background

### The discovery of nitric oxide

Nitric oxide (NO) was the first endogenous gaseous signalling molecule to be discovered and was the product of work undertaken over an extended period by many scientists tackling questions about vascular and immune cell function. The culmination of this work included the demonstration by Robert Furchgott that rapid reductions in smooth muscle tone were driven by production of an endothelial cell dependent mediator. Described initially as endothelial derived relaxing factor (EDRF) [1], Salvador Moncada went on to demonstrate that EDRF was in fact identical to NO [2]. In parallel, the discovery by Ferid Murad that nitric oxide (NO) was a potent activator of soluble guanylate cyclase [3] ultimately led to the confirmation by Louis Ignarro that NO was the second messenger molecule responsible for reducing smooth muscle tone through this mechanism. This work secured Furchgott, Ignarro and Murad the Nobel prize for physiology or medicine in 1998 [4], an award that was built on a body of work going back many years across many disciplines.

### NO synthesis and actions

NO is synthesised by nitric oxide synthase (NOS) from the guanidino group of arginine through oxygen and NADPH dependent oxidation producing NO, with citrulline as a by-product. Three isoforms of nitric oxide synthase regulate the production and vary in their tissue distributions. Neuronal NOS (nNOS) is predominantly found in the nervous and enteric systems but also in vascular smooth muscle [5] and cardiac myocytes [6]. Endothelial NOS (eNOS) is found predominantly in the endothelium and cardiac mycoytes [7]. The inducible isoform (iNOS) is widely expressed in response to inflammatory stress although is found constitutively at low levels in some tissues.

The actions of NO can be broadly divided into direct and indirect. Classically, NO acts as a second messenger through direct activation of soluble guanylate cyclase (sGC) resulting in increased synthesis of cyclic guanosine monophosphate (cGMP) and reduced vascular tone. In addition to the direct action on sGC, NO directly binds to heme moieties on a range of proteins and leads to a number of processes including modulation of the mitochondrial electron transport chain at the level of complex IV and inhibition of cytochrome P450–mediated metabolism.

Indirectly, NO mediates its effects through its role as a free radical. NO rapidly interacts with other free radicals such as superoxide to form secondary metabolites such as peroxynitrite (ONOO^−^). Through similar reactions it can intercept lipid oxidation products (peroxy radicals) and act as chain-breaking antioxidant [8, 9].

The impact of the NO production in sepsis is extensive; however, the literature and in some respects its interpretation are conflicting. A detailed review of the function of NO is beyond the scope of this review; however, NO is essential to the maintenance of normal cardiovascular and immune responses to infection. It has diverse effects including maintenance of microvascular function, regulation of platelet aggregation and leukocyte activity, adhesion and transport [10]. Similarly, NO is directly toxic to most bacteria and has free radical scavenging action which reduces tissue injury and bioenergetic dysfunction. However, in sepsis and septic shock, these processes become dysregulated to variable degrees. Exaggerated NO production has been implicated in the development of macrovascular compromise [11], myocardial dysfunction [12], reduced responsiveness to adrenergic stimuli [13, 14], direct cellular toxicity and bioenergetic failure [15, 16]. Conversely, impaired local NO production in the endothelium has been cited as a cause of the microvascular dysfunction and impaired regional perfusion seen in septic shock.

### Regulation of NO synthesis

The balance of harmful and beneficial effects is determined by a series of regulatory processes that determine the site and degree of NO synthesis that arises in response to an inflammatory stimulus.

**Fig. 1:**
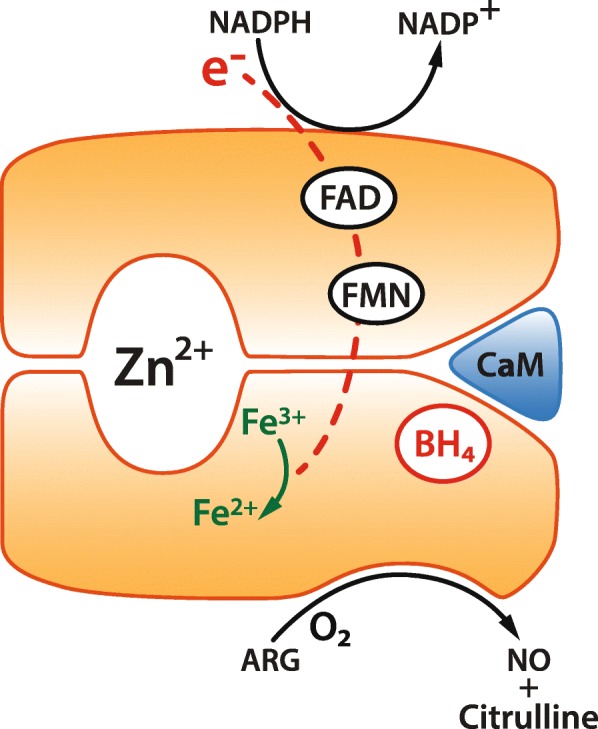
Nitric oxide synthase (NOS) cofactors and activity. NOS enzymes function as homodimers within subunits structurally supported by a Zinc ion (Zn^2+^). Following sequestration of iron, the binding site for arginine is expressed. Electron transfer also depends on flavin mononucleotide (FMN) and flavin adenine dinucleotide (FAD) availability and the binding of calmodulin (CaM) which is calcium dependent for eNOS and nNOS and independent for iNOS. NADPH, oxygen and tetrahydrobiopterin (BH4) are essential cofactors for the synthesis of nitric oxide

The active isoforms of NOS are homodimers and depend upon a range of co-factors for their activity. Dimerisation is essential for activation and leads to the sequestration of iron and creation of binding sites for arginine and the essential cofactor tetrahydrobiopterin (BH4). In addition, dimerisation facilitates electron transfer between two key domains linked by a calmodulin binding motif. In eNOS and nNOS, the binding of calmodulin to the NOS homodimer is calcium dependent. Conversely, calmodulin is tightly bound to iNOS and therefore displays calcium independent activation. Enzymatic activity also depends upon, haem, flavin mononucleotide (FMN) and flavin adenine dinucleotide (FAD) availability as well as oxygen and NADPH as co-substrates (Fig. 1).

Differential expression of the NOS isoforms within the cell or in tissues also influences NO synthesis. eNOS is commonly found bound to caveolin, a specialised anchoring protein within the caveolae of the endothelial cell membrane [17] which may itself regulate NO production [18]. This conformation has been implicated in the development of reduced NO synthesis in the septic microvasculature. In myocytes, the three NOS isoforms are differentially distributed with eNOS predominantly found in endothelium, nNOS in the presynaptic nerves and both found in different portions of the sarcoplasmic reticulum. iNOS by contrast, when induced by pro-inflammatory stress is most commonly found in the cytoplasm where it generates a two or three log fold higher increases in NO synthesis compared to the constitutive isoforms.

## Therapeutic modulation of NO in sepsis

In the years following its discovery came a burgeoning recognition that NO played an important role in the development of the vasoplegia and hypotension that is the hallmark of septic shock. This led first to small scale studies of inhibition of sGC activity by methylene blue [19, 20], and subsequently, clinical trials of the non-specific small molecule inhibitor of all three NOS isoforms, L-NMMA. Phase II results [21] showed promise as L-NMMA demonstrated improved systemic vascular resistance and blood pressure in patients with septic shock. However, a phase III trial was terminated early due to excess mortality in the treatment group [22]. Questions regarding dose selection, blood pressure management and treatment strategy meant that interest in the area was not extinguished, and subsequent work has recognised the plethora of roles that NO has in regulating organ function in both health and disease.

In recent years, potential approaches to modulating NO production in sepsis have been developed targeting pathways beyond direct NOS inhibition. The remainder of this review will focus on a selection of these strategies which include modulating endogenous regulators of NOS activity, NO scavenging or donation and optimising substrate availability.

## Nitric oxide inhibition

### Endogenous regulators of NOS activity

Tetrahydrobiopterin (BH4) is an essential cofactor for NOS-mediated NO synthesis through facilitation of electron transfer to L-arginine. Reduced BH4 bioavailability results in the diversion of electron flow to molecular oxygen and production of free radical superoxide rather than N O[23], a process also known as NOS uncoupling. Reduced BH4 bioavailability has been implicated in the development of chronic cardiovascular diseases through this mechanism.

Whilst in sepsis, pro-inflammatory stress has been shown to increase production of BH4 [24], coexistent oxidative stress and reactive oxygen species cause conversion of BH4 to BH2. In addition to depleting BH4 scores, BH2 binds to NOS with equal affinity but promotes the uncoupling process [25] thereby reducing NO production. In malaria, increasing severity of disease is associated with reduced BH4 levels and BH4:BH2 ratio [26] and has been proposed as a driver of microcirculatory dysfunction in this population.

Supplementation of a BH4 analogue appears to reduce haemodynamic impairment, organ dysfunction and improve survival in rat models of acute inflammation and sepsis [27–29]. In an ovine model of septic shock secondary to faecal peritonitis, supplemental tetrahydrobiopterin therapy was associated with preservation of the sublingual microcirculation, reduced cardiovascular compromise and severity of lung and renal injury [30]. Furthermore, In animal models, ascorbate supplementation has been shown to restore capillary blood flow and eNOS activity in a BH4 dependent manner [31]. This has been postulated as one of the mechanisms underlying the potential benefits of Vitamin C supplementation in sepsis.

A further example of potential therapeutic approaches includes increasing the expression of endogenous inhibitors of NO synthesis. Derived from the methylation of arginine residues by the Protein Arginine Methyl Transferases (PRMTs), the methylarginines have an unusual physiological role in regulating the synthesis of NO. The three methylarginines are asymmetric dimethylarginine (ADMA), symmetric dimethyl arginine (SDMA) and, occurring at approximately a tenth of the concentration, monomethylarginine (L-NMMA). The asymmetric pair, ADMA and L-NMMA, acts as equipotent competitive inhibitors of arginine binding to the all three NOS isoforms. By contrast, SDMA has no direct action on NOS activity. Elevated levels of both ADMA and SDMA are associated with poor outcome in patients with sepsis; however, an increase in the ratio of ADMA to SDMA is associated with reduced mortality in patients with septic shock [32], suggesting there may be a disconnection between the association of elevated ADMA levels and the mechanisms responsible for dysregulated NO production.

**Fig. 2:**
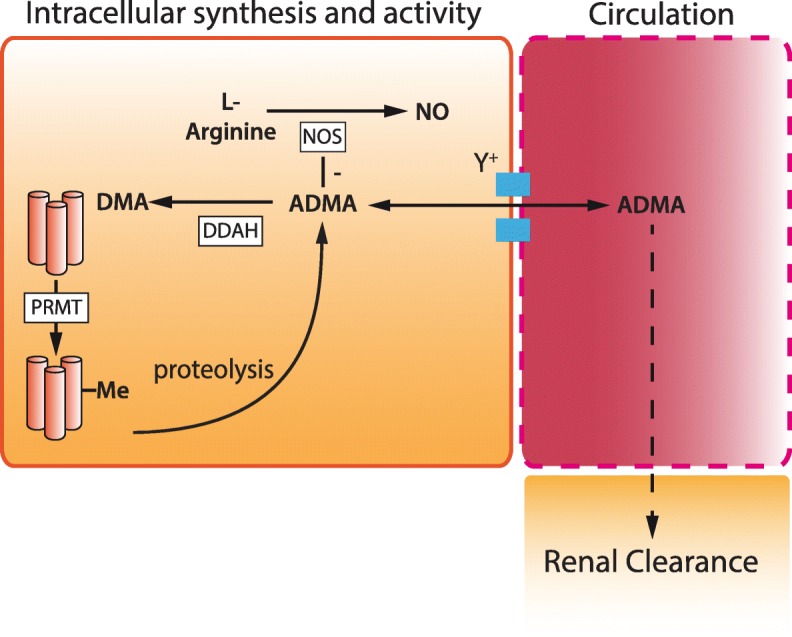
Methylarginine-mediated nitric oxide synthase inhibition. Representative image of the synthesis and regulation of ADMA. Protein Arginine Methyl Transferases (PRMT) catalyse the methylation of arginine containing protein residues to ADMA and SDMA which are released upon proteolysis. ADMA and SDMA are transported via the y^+^ cationic amino acid transporter into and out of the circulation. ADMA is metabolised by the two isoforms of dimethylarginine dimethylaminohydrolase (DDAH) in a wide range of tissues. ADMA acts intracellularly to inhibit nitric oxide synthase (NOS). ADMA is largely metabolised by DDAH to dimethylamine (DMA), a small amount is cleared unchanged through the kidney. nb. L-NMMA (monomethylarginine) is considered to have the same synthetic pathway, activity, metabolism and clearance as ADMA but is present in only 10% of the concentration

The dominant method of methylarginine handling is enzymatic and driven by one of the two dimethylarginine dimethylaminohydroxylases (DDAH1 and DDAH2). Approximately 80% of ADMA and L-NMMA are metabolised to dimethylarginine (DMA) and L-citrulline by the DDAH isoforms, with the remaining 20% excreted by the kidney [33] (Fig. 2).

In addition to expression in the vasculature [34], DDAH1 is readily identified in the liver, kidney, brain, skeletal muscle and pancreas [35]. Small amounts of DDAH1 have also been found in the pulmonary vasculature [36] and in placental [37] tissues. The tissue distribution of DDAH2 differs markedly from that of DDAH1. Whilst both isoforms are found in blood vessels, liver and kidney, DDAH2 predominates in the placenta and is not found in the central nervous system. It is also the only isoform found in cardiomyocytes and in immune tissues [34, 35, 38]. This has led to the suggestion it may play an important role in the response to inflammatory or immune mediated conditions.

In rodent lipopolysaccharide (LPS) and polymicrobial models, both genetic and therapeutic inhibition of DDAH1, has been shown to offer improved survival [39]. In addition, DDAH1 inhibitor therapy was found to reduce the severity of hypotension and requirement for noradrenaline to maintain normal blood pressure. These effects still occurred when treatment was delayed until after the onset of shock symptoms. Furthermore, DDAH1 inhibition was shown to improve indices of renal and hepatic function, preserve microvascular flow and improve survival without any effect on immune cell function [39]. This favourable balance of improved vascular function without the deleterious impacts on the cardiac or immune system has led to DDAH1 inhibition being postulated as a potential therapeutic approach in septic shock.

### Nitric oxide scavenging

The recognition that NO has essential functions in sepsis and that global inhibition of NO synthesis could have harmful effects, led to the hypothesis that scavenging excess NO might prove an effective therapeutic strategy [40]. One of these approaches was the development of pyridoxylated haemoglobin polyoxyethylene (PHP), which was originally developed as a candidate oxygen delivery substitute. PHP is pyridoxylated to remove its oxygen carrying capacity and then combined with polyoxyethylene to increase the apparent molecular weight of the compound. The resulting product has a high affinity for NO which it rapidly oxidises to the more stable nitrate as well as reducing peroxynitrite concentrations and regulating the redox state of the cell [41].

Preclinical ovine models of sepsis demonstrated that PHP improved systemic vascular resistance [42] and that treatment was not associated with impaired tissue perfusion. This led to a phase II study in of 62 patients with septic shock [43]. In this study, those treated with PHP displayed trends towards reduced requirements for vasopressors and ventilatory support. This was followed by the phase III PHOENIX study [44], which recruited 377 patients. The study showed no survival benefit following PHP therapy at 28 days but evidence of increased mortality in patients with a SOFA score of > 13 on inclusion. Despite this, the previously observed improvements in systemic haemodynamics persisted, with survivors displaying reduced duration of vasopressor therapy. The authors concluded that regional variation in NO synthesis in the microcirculation could have been exacerbated in the PHP treated patients thereby worsening organ perfusion and accounting for the worse outcomes observed. This coupled with the inability to identify which patients had more substantially elevated systemic NO levels based on noradrenaline requirement meant that selecting patients that might benefit from this approach based on clinical indices was not possible.

## Increasing nitric oxide bioavailability

### Substrate bioavailability

Arginine is the substrate for NOS enzymes and its availability is a dominant factor in the regulation of NO production. Whilst most bioavailable arginine is derived from the diet, the intracellular concentrations are governed by a range of processes including transmembrane transport, enzymatic synthesis and metabolism.

In sepsis, arginine becomes a conditionally essential amino acid with reduced bioavailability resulting from a variety of factors. Exaggerated protein turnover leads to an increase in stored arginine release however in patients with sepsis, reduced dietary intake of arginine and citrulline [45, 46] is coupled with increased turnover by NOS and arginase enzymes [47]. This coupled with reduced de novo synthesis at the tissue level in organ failure [48] means that circulating arginine concentrations are substantially reduced regardless of the infectious cause [49–51] with direct consequences for the production of NO and the microcirculation [52]

Extensive investigation has been undertaken exploring the potential role of exogenous arginine supplementation in sepsis. A number of animal models have produced promising results with a murine [52] and pretreatment porcine inflammatory model [53] showing improved microvascular function and organ perfusion mediated by increased NO production. In healthy volunteers, arginine supplementation is well tolerated at doses less than 8g/day; however, supplementation at doses above this was associated with gastrointestinal side effects [54]. In patients with critical illness, arginine supplementation has not consistently demonstrated clinically relevant improvements in outcome. There is some trial evidence suggesting supplementing arginine in addition to other nutrients may result in a reduced hospital length of stay [55] or improved organ function [56]. Preiser et al. [57] successfully increased plasma arginine concentrations via enteral supplementation in a small randomised controlled trial of a mixed ICU population. Whilst this was associated with some signal of increased arginase activity, no increase in plasma markers of NO production was detected. In a subsequent randomised trial of 597 critically ill patients, an immunonutrition approach including supplementation of arginine as well as glutamine and omega-3 was not associated with improved clinical outcomes [58]. A further study of arginine containing enteral immunonutrition versus parenteral therapy was stopped after an interim analysis suggested an excess mortality in patients receiving enteral supplementation [59]. This has led to the recommendation in the surviving sepsis guidelines that arginine supplementation is not used to treat patients with sepsis [60].

**Fig. 3:**
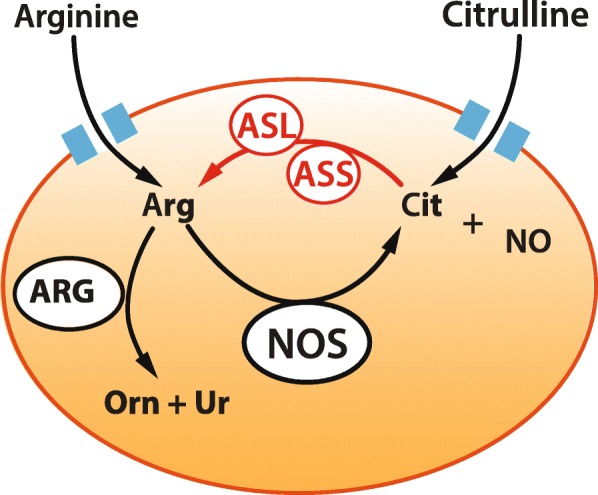
Arginine bioavailability. Arginine (ARG) is transported into the cell via cationic amino acid transporters and is converted to citrulline (Cit) and NO. Citrulline derived from a number of sources including diet can be converted to arginine by argininosuccinate synthetase (ASS) and argininosuccinate lyase (ASL) increasing the available intracellular arginine. Arginase (ARG) metabolises arginine to ornithine (Orn) and urea (Ur)

In addition to dietary uptake, arginine can be synthesised through the conversion of citrulline by a pair of enzymes that are found in the cytosol, argininosuccinate synthetase (ASS) and argininosuccinate lyase (ASL) [61] (Fig. 3). The majority of bioavailable citrulline is synthesised by enterocytes with some derived from the diet [62] and a small proportion synthesised in the kidney, liver and enterocytes from ornithine by ornithine transcarbamoylase [63]. Citrulline bioavailability is also reduced in sepsis. Observed in patient populations to be associated with increased mortality [64], a low citrulline concentration is likely multifactorial. In addition to reduced dietary intake, reduced uptake of the citrulline precursor glutamine from the gut may play a role [65]. Compensatory increases in the expression of ornithine transcarbamoylase which synthesises citrulline from ornithine in the kidney may mitigate this effect to some degree [66].

A number of cell types use citrulline conversion by ASS and ASL to generate endogenous arginine. These include vascular smooth muscle [67], macrophages [68], neural cells [69] and endothelial cells [70, 71]. Importantly, in endothelial cells, ASS and ASL co-locate with eNOS and play an important role in regulating endogenous NO synthesis [72].

In sepsis, ASS expression is elevated in both the vasculature and immune cell populations, possibly in order to facilitate local NO production by iNOS [73, 74]. In animal models, heterozygote and tissue specific knockout of ASS are associated with worse outcomes due to reduced immune cell NO synthesis [75]. Supplementation with recombinant ASS mitigates the lethal effects of LPS in animal models although the mechanism of efficacy may also be related to direct binding to Gram negative bacteria reducing organism pathogenicity rather than the production of increased arginine [74].

With the similar aim of augmenting arginine and subsequent NO bioavailability and with some evidence from animal models [52], citrulline supplementation has also been proposed as a possible treatment strategy in sepsis. Whilst there is some evidence of association between low citrulline concentrations and the development of ARDS [46] and gut mucosal integrity in patients with sepsis [76], there is no prospective evidence to recommend citrulline supplementation at this stage.

The isoforms of the arginase enzymes degrade arginine to ornithine and urea rendering it an important regulator substrate availability and therefore of NO synthesis in the cell (Fig. 3). The arginase isoforms have differing tissue distributions, with arginase 1 expressed in endothelial cells, immune cells and the liver. Arginase 2 is also found in the endothelium [77] and immune cells [78] but is also detected in the kidney, brain and small intestine.

The expression of arginase isoforms during sepsis is a dynamic process that points to a role in regulating the initiation and resolution of inflammation [79–81] by limiting arginine availability. After an acute inflammatory stress, arginase induction typically arises some hours after peak iNOS induction [45]. This is responsible in part for the downregulation of NO synthesis and is best characterised in immune cells where it defines the transition of the macrophage from the pro-inflammatory M1 to the restorative M2 phenotype where arginase mediates the synthesis of proline and polyamines crucial to the process of wound healing [82].

Arginase 1 knockout immune cells and mice deficient in arginase 1 in endothelial and haematopoietic cells are prone to exaggerated NO synthesis in LPS models of inflammation with an associated microvascular dysfunction seen in murine models [83]. In patients, limited data suggests that levels of the enzyme in the circulation are elevated in sepsis but is not directly related to clinical indices of illness severity [84]. Although no therapeutic evaluation of arginase modulation is underway, the role of the enzymes in regulating systemic and local arginine concentrations is undoubtedly important and further understanding of the balance between NOS and arginase expression may yield novel therapeutic approaches.

### Targeting microcirculatory dysfunction

The wide recognition of the importance of NO in maintaining the microcirculation, combined with the negative outcomes of trials of NOS inhibition has also led to suggestions that rather than reducing synthesis of NO, attention should focus on the use of NO donors to improve microvascular function, reduce shunting and optimise tissue perfusion in septic shock. Two distinct approaches have been proposed. The first involves optimising eNOS activity within the vascular endothelium which, as described above, is known to be impaired in sepsis due to the uncoupling of NO production due in part to reduced BH4 availability, lack of availability of substrate and elevated oxidative stress.

The alternative therapeutic strategy is to deliver exogenous compounds which can release NO at the microcirculatory level in order to optimise small vessel function. There are a number of proposed functions of the microcirculation that could be improved through NO modulation which include smooth muscle relaxation, reduced platelet aggregation and microthrombus formation and reduced endothelial leak and interstitial oedema. In addition, there is evidence that inorganic nitrites may offer some protection from ischaemia reperfusion injury and have a cardioprotective effect through a range of actions on mitochondrial and cellular function [85, 86].

This approach was tested in a small study of 8 patients as early as 2002 with nitroglycerin infusion which led to reported improvements in the sublingual microcirculation [87] although a subsequent randomised controlled trial published in 2010 randomised 70 patients to receive nitroglycerin infusion or placebo and found that this was not associated with improved microvascular flow in any category of vessel or interestingly on systemic haemodynamics, suggesting that it may not have been having the desired pharmacodynamic effect [88]. One potential reason for this could be the failure to effectively convert nitroglycerin to NO in vivo due to dysfunction of aldehyde dehydrogenase type 2 in capacitance vessels, which may not function effectively in patients with sepsis and leads to reduced nitroglycerin bioactivation [89]. This however remains only one endogenous route of activation [90] and therefore may not entirely explain this apparent result.

Based on a similar methodological approach, the direct administration of NO via an inhaled route has recently been trialled in patients with sepsis [91]. In a randomised trial of 50 patients, direct NO administration was able to increase circulating nitrite levels consistent with the hypothesis that pulmonary delivery of NO could lead to increased systemic availability; however, no impact on sublingual microvascular or organ function was detected.

## Conclusions

Extensive research has led to the recognition that NO synthesis is regulated by a range of mechanisms which determine the production and actions of NO. Substrate and co-factor availability all contribute to NOS activity, which also determined on a tissue level by protein expression, cellular localisation and the presence of endogenous regulators.

In the last decade therapeutic research into the modulation of NO in critical illness has taken on a Janusian quality as it explores the competing challenges of overproduction of NO in some systems and impaired synthesis in others. Exaggerated NO production is responsible for cardiac, macrovascular and cellular dysfunction; however, microvascular function due to reduced eNOS activity and NO synthesis also appears to play an important role in outcome. The development of novel treatments in this area must mitigate the pathological consequences of at least one of these without exacerbating the other or impairing the essential physiological NO synthesis that is core to the response to infection. This challenge must be addressed if an improved understanding of the mechanistic role of NO in sepsis can be translated into therapies that improve outcomes for patients.

## Data Availability

Not applicable

## Abbreviations

ADMA: Asymmetric dimethylarginine
ASL: Arginosuccinate lyase
ASS: Arginosuccinate synthetase
BH4: Tetrahydrobiopterin
cGMP: Cyclic guanosine monophosphate
DDAH: Dimethylaminohydroxylases
DMA: Dimethylarginine
EDRF: Endothelial derived relaxing factor
FAD: Flavin adenine dinucleotide
FMN: Flavin mononucleotide
L-NMMA: Monomethylarginine
LPS: Lipopolysaccharide
NO: Nitric oxide
NOS: Nitric oxide synthase
PHP: Pyridoxylated haemoglobin polyoxyethylene
PRMTs: Protein Arginine Methyl Transferases
RNS: Reactive nitrogen species
SDMA: Symmetric dimethyl arginine
sGC: Soluble guanylate cyclase
SOFA: Sequential Organ Failure Assessment
